# Lactoperoxidase potential in diagnosing subclinical mastitis in cows via image processing

**DOI:** 10.1371/journal.pone.0263714

**Published:** 2022-02-17

**Authors:** Emmanuelle P. E. Silva, Edgar P. Moraes, Katya Anaya, Yhelda M. O. Silva, Heloysa A. P. Lopes, Júlio C. Andrade Neto, Juliana P. F. Oliveira, Josenalde B. Oliveira, Adriano H. N. Rangel

**Affiliations:** 1 Postgraduate Program in Animal Production, Federal University of Rio Grande do Norte, Macaíba, Rio Grande do Norte, Brazil; 2 Institute of Chemistry, Federal University of Rio Grande do Norte, Natal, Rio Grande do Norte, Brazil; 3 Faculty of Health Sciences of Trairi, Federal University of Rio Grande do Norte, Santa Cruz, Rio Grande do Norte, Brazil; 4 Academic Unit Specialized in Agricultural Sciences, Federal University of Rio Grande do Norte (UFRN), Macaíba, Rio Grande do Norte, Brazil; 5 Rural Health and Technology Center, Federal Rural University of Campina Grande, Patos, Paraíba, Brazil; INRAE Centre Val de Loire: Institut National de Recherche pour l’Agriculture l’Alimentation et l’Environnement Centre Val de Loire, FRANCE

## Abstract

This report describes how image processing harnessed to multivariate analysis techniques can be used as a bio-analytical tool for mastitis screening in cows using milk samples collected from 48 animals (32 from Jersey, 7 from Gir, and 9 from Guzerat cow breeds), totalizing a dataset of 144 sequential images was collected and analyzed. In this context, this methodology was developed based on the lactoperoxidase activity to assess mastitis using recorded images of a cuvette during a simple experiment and subsequent image treatments with an R statistics platform. The color of the sample changed from white to brown upon its exposure to reagents, which is a consequence of lactoperoxidase enzymatic reaction. Data analysis was performed to extract the channels from the RGB (Red-Green-Blue) color system, where the resulting dataset was evaluated with Principal Component Analysis (PCA), Multiple Linear Regression (MLR), and Second-Order Regression (SO). Interesting results in terms of enzymatic activity correlation (R^2^ = 0.96 and R^2^ = 0.98 by MLR and SO, respectively) and of somatic cell count (R^2^ = 0.97 and R^2^ = 0.99 by MLR and SO, respectively), important mastitis indicators, were obtained using this simple method. Additionally, potential advantages can be accessed such as quality control of the dairy chain, easier bovine mastitis prognosis, lower cost, analytical frequency, and could serve as an evaluative parameter to verify the health of the mammary gland.

## Introduction

Mastitis is an endemic disease, which affects the mammary glands, is characterized by an inflammatory response involving metabolic, physiological, and traumatic changes caused by pathogenic microorganisms [1]. The condition causes great economic losses in the agricultural sector, causing a decrease in milk production and quality, and results in its disposal, treatment costs, reduced longevity, and an increase in the slaughter rate of the animals [2–4].

Mastitis can cause changes in the ion, protein, and somatic cell concentrations, as well as in some enzymes which can affect the yield and quality of dairy products [5,6].

In this context, lactoperoxidase (LPO) is one of the enzymes which are active in this process. It acts in the defense of the mammary gland through the lactoperoxidase system during the initial infection stages by producing antimicrobial substances, which in turn act by destroying or inhibiting the action of pathogens [7,8]. Research shows that LPO may be able to help in diagnosing subclinical mastitis, as cows that have a high somatic cell count also have increased enzyme activity [9].

Mastitis control has been constantly investigated and most studies focus on vaccine development or herd management to reduce or eliminate the causative agents. The current focus of investigating the disease addresses modulating the immune response of the mammary gland to increase the gland’s resistance to invading pathogens since the udder immunity is also responsible for the clinical outcome of the disease [4].

In recent years, computational tools in artificial intelligence, such as machine learning, are being successfully applied in diverse areas. Significant progress has been achieved in imaging recognition, including methods for monitoring animal health conditions [10]. Deep convolutional neural networks have powerful machine-learning functions on large-scale training datasets, achieving significant achievements in target detection scenarios with complex backgrounds. These techniques include the detection of animal disease [10].

Imaging analysis by thermal infrared was capable of detecting mastitis in dairy cows through the correlation between infrared image and somatic cell count (SCC). The accuracy of the mastitis classification algorithm was 83.33%, the sensitivity was 92.31% and the specificity was 76.47%. This method realized the accurate positioning of key parts of dairy cows and can be used for the automatic recognition of dairy cow mastitis [11].

Colorimetric detection from digital image capture presents instrumental simplicity and has been explored in various analytical applications. Digital image capture devices such as smartphones offer some advantages, including accessibility, portability, low cost, and battery power or USB (Universal Serial Bus) connection with portable computers. These advantages provided by digital image capture devices encourage the use of this instrumental tool for remote analysis or in places where the availability of resources is limited. These factors favor the use of portable systems to conduct remote analyses, in which a preliminary result, even if qualitative, can be essential for a preventive diagnosis [12–14].

Furthermore, digital image processing can refine the visual quality of the data by reducing noise, improving the detection, and, most importantly, providing a more reliable analysis. This strategy has been used in multivariate image analysis methods like principal component analysis, allowing classification (or discrimination), segmentation defect detection, and even prediction [12]. Thus, in being aware of the growing need to ensure the quality of milk produced and aiming to contribute to developing new tools to diagnose bovine mastitis, we sought to identify and quantify the lactoperoxidase activity in milk samples from cows. In addition, to further evaluate the potential of the lactoperoxidase system and somatic cell count using an accessible and economical method by image analysis to detect mastitis in dairy cows.

## Material and methods

The project was submitted to evaluation by the Ethics Committee on the Use of Animals at UFRN (protocol No. 051.061/2017) and all animal management practices followed the recommendations of the National Council for the Control of Animal Experimentation (CONCEA) for the protection of animals used for animal experimentation and other scientific purposes.

The sample universe was represented by one (n) group of 48 samples of cow’s milk, with 32 samples coming from Jersey animals obtained from the São Miguel farm located in São Gonçalo do Amarante, RN, Brazil; 07 samples from Gir animals and another 09 from Guzerat from the Felipe Camarão Experimental Station (EMPARN)/Parnamirim, RN, Brazil. The samples were taken in triplicate from the milking system collectors and identified with the animal’s code in polyethylene bottles of approximately 40 mL. No type of preservative was used. They were packed in isothermal boxes with ice at a temperature between 2°C and 6°C and subsequently sent to the Milk Quality Laboratory (LABOLEITE/UFRN) for analysis.

### Analytical methods

The samples were subjected to somatic cell count analysis (SCC) using the Somaticell^®^ Kit (Idexx Laboratories Inc., Westbrook, Maine, USA), qualitative lactoperoxidase determination by the methodology proposed by the Ministry of Agriculture, Livestock and Supply (MAPA) [15]. There were performed together with image acquisition of the samples submitted to a qualitative lactoperoxidase determination for later quantification analysis by the RGB color system (Red-Green-Blue).

### Somatic cell count

The samples were subjected to somatic cell count analysis using the Somaticell^®^ Kit which has the operation objective to determine the somatic cell count from the viscosity of milk against a specific reagent in a direct proportion, in which the more viscous the milk, the more somatic cells it presents. The sample reading was performed according to grading the analysis flask which varied from 69 to 1970. The number in the tube at the milk level indicated the estimated amount of somatic cells in thousands.

### Qualitative lactoperoxidase determination

The reagents used to qualitatively analyze the enzyme were 1% guaiacol and 10 Vol hydrogen peroxide. First, 10 mL of the milk aliquot was added to a test tube. The sample was subjected to heating at 45°C in a water bath for 5 minutes for enzymatic activation. After that period, 2.0 mL of 1% guaiacol and 3 drops of 10 Vol hydrogen peroxide. A positive result was the formation of a solution in salmon color varying between light and dark after 5 minutes. Then, the samples were transferred to a quartz cuvette to be photographed and analyzed.

### Data acquisition of the samples

**Fig 1:**
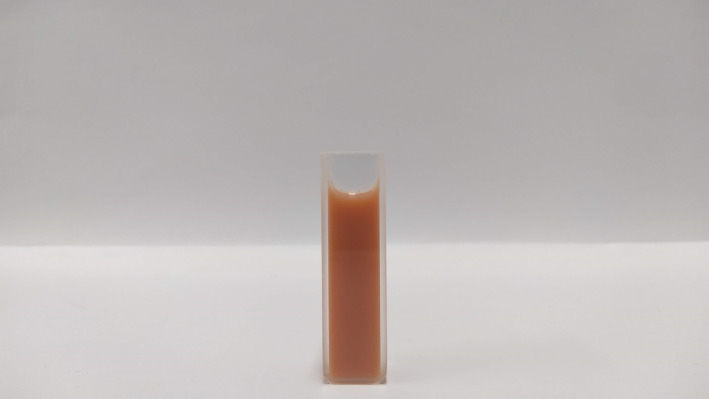
Cuvette image of the milk sample treated. Example of capture strategy using a common smartphone.

The quartz cuvettes with the samples were placed on a carton apparatus with opaque white bottom and background (Fig 1). Imaging acquisition was performed with a regular smartphone 12 megapixels resolution camera (Motorola G5S, Motorola Mobility, Brazil), in automatic mode and with no filters. The cuvette was placed 10 cm of distance from the White background as well as 10 cm from the camera (attached to a tripod). Imaging acquisition was performed in triplicate and under the same lighting conditions in the room. A dataset of 144 sequential images was collected and analyzed.

### Quantitative lactoperoxidase determination

A 5100PC UV/VIS spectrophotometer (São Paulo, BR) was used at an absorbance of 412 nm as a function of time for two minutes to determine the enzymatic activity in milk samples. The milk samples were diluted to 1:250 in sodium phosphate buffer solution pH 7.0. Volumetric flasks of 10 mL were used in which 40 μL of milk were needed for this dilution. Each sample had its respective blank and 0.1 ml of milk in solution and 3.0 ml of ABTS solution were added to a quartz cuvette for reading. Next, 0.1 mL of the milk in solution, 3.0 mL of the ABTS 0.001 mol L^-1^ solution, and 0.1 mL of the 0.0032 mol L^-1^ hydrogen peroxide solution were added to the cuvette to read the sample. The reading was performed immediately and the initial absorbance value and the value after two minutes were recorded, with the change in absorbance value in that time interval being used for the enzymatic concentration calculations. The results were expressed in unit of enzymatic activity/mL, for which a unit of enzymatic activity was defined as the amount of enzyme that catalyzes the oxidation of 1μmol of ABTS per minute at room temperature. The enzymatic activity was calculated according to [16].

### Calibration curve

A pure bovine lactoperoxidase enzyme from Sigma-Aldrich (Saint Louis, USA) was used to obtain the calibration curve for the lactoperoxidase enzyme. Therefore, 1 mg of the lactoperoxidase enzyme was used and dissolved in 1 liter of the pH 7.0 phosphate buffer solution. Next, 1.0 to 9.0 mL of the enzyme in solution was manipulated and dilutions ranging from 100 to 900 μL were performed using 10 mL volumetric flasks. The method for reading the samples was similar to that performed with milk samples.

### Data processing and chemometric treatment

Data analysis was performed using the R 4.1.1 statistical platform. Median filtering (EBImage package) was applied in pre-processing, which reduces the amount of intensity variation between neighboring pixels in the images. The separation of RGB matrices (EBImage package) was performed by calculating the isolated color intensity for each channel as an algebraic average and the colorimetric absorbance analytical response (A_c_) was calculated using the equation below as an approximation to the Lambert-Beer Law:

$$A_{c}=‐\log\;I/I_{0}$$ (1)

In which:

I*—*Mean intensities of one of the RGB components for the region of the functionalized sample; and *I*_0 *−*_Non-functionalized region.

**Fig 2:**
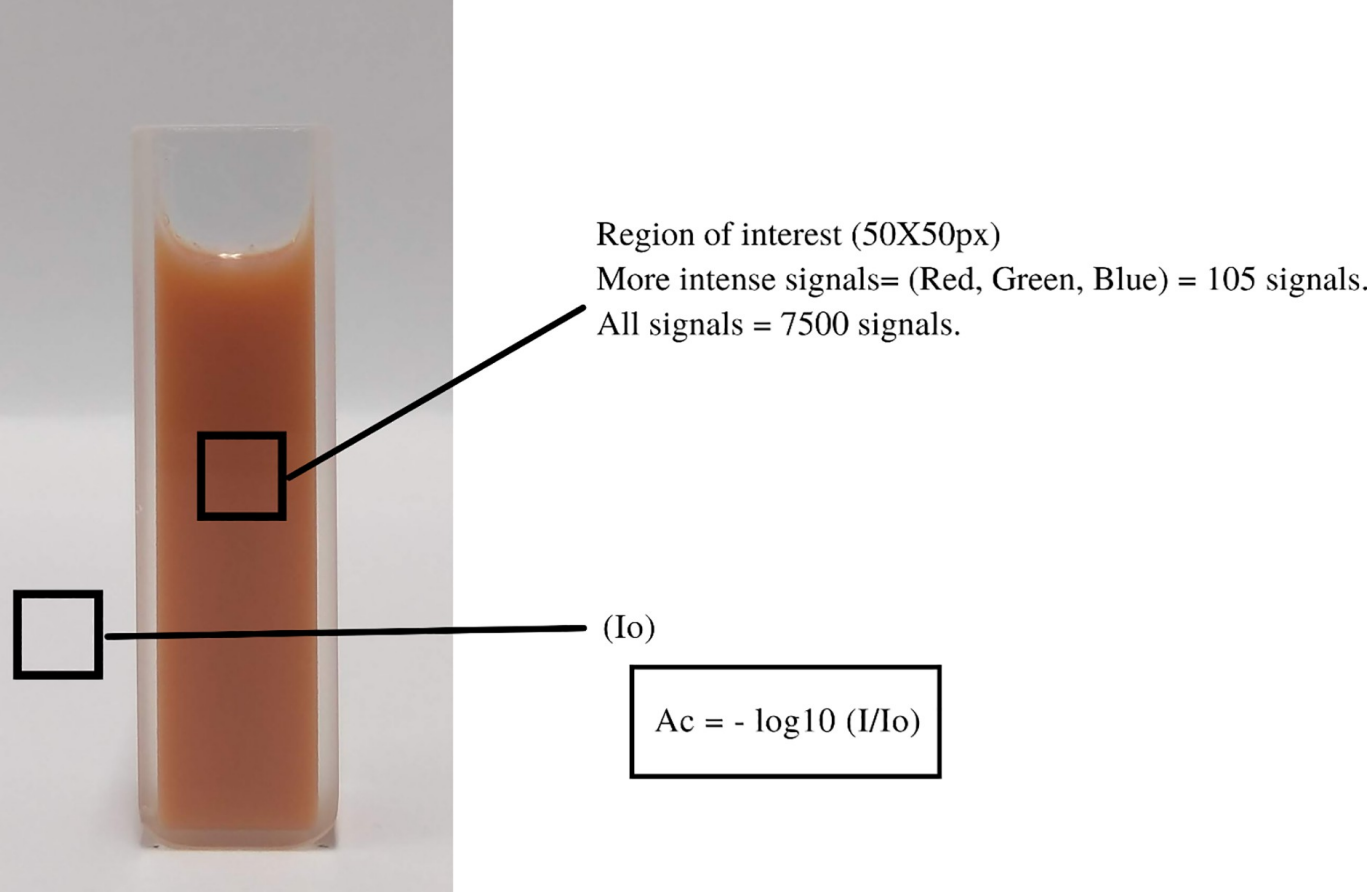
Spots of the region of interest (ROI) for image processing. Here we denote the 2 ROIs as the blank (left) and sample (right).

The regions of interest (ROIs) in the images were selected using the free software GIMP 2.8.18. Square portions of 50×50 pixels were chosen from the center of each region of the cuvette (see Fig 2) and recorded in JPEG format.

PCA analysis (Chemometrics with R^6^ and factoextra^7^ package) for the image data of the samples was developed using the absorbance values resolved for the RGB channels (normalized via auto-scaling).

## Results and discussion

### Calibration curve

**Fig 3:**
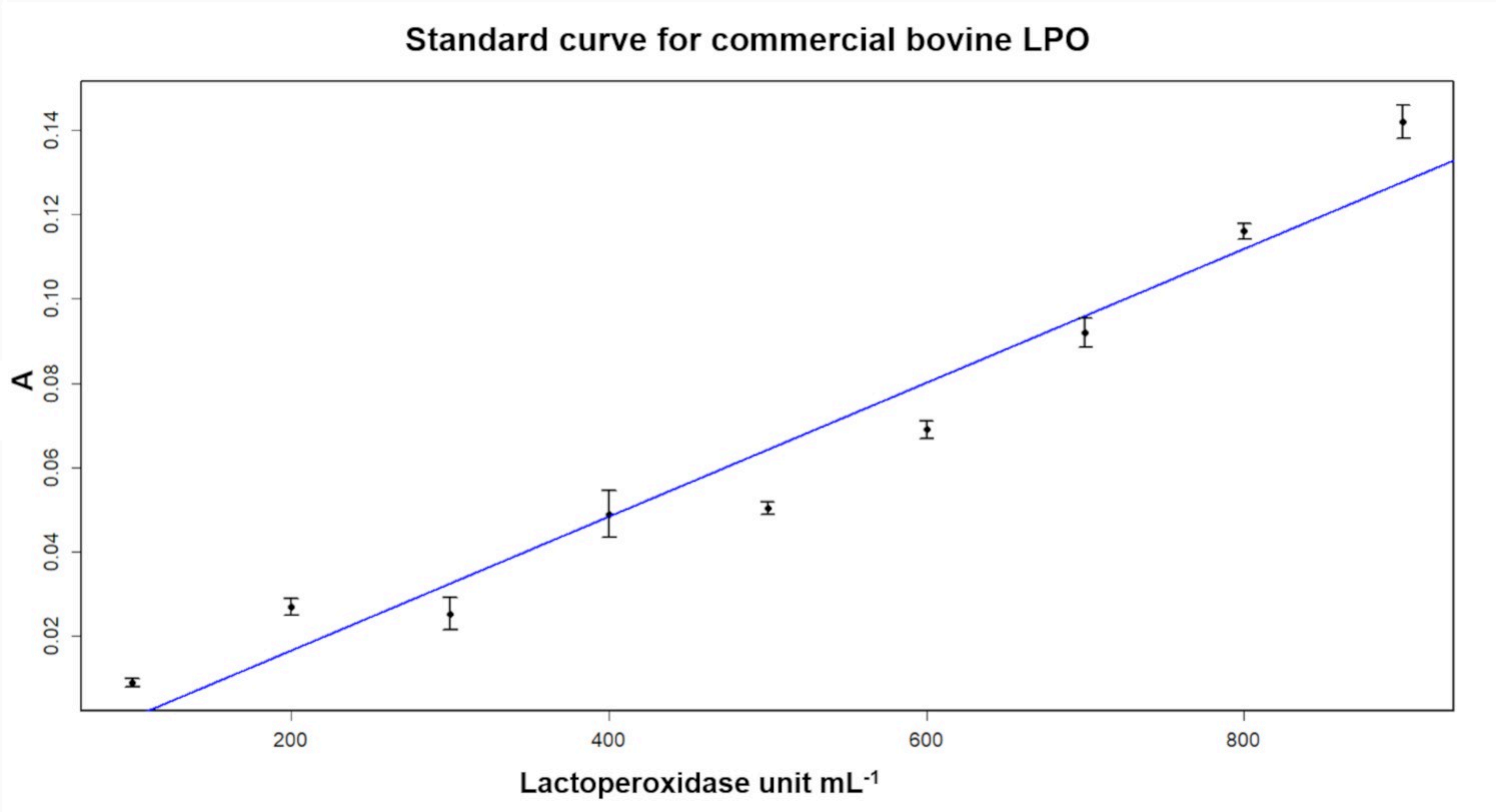
LPO calibration curve in units mL^-1^. Calibration curve for lactoperoxidase analysis showing the absorbance vs lactoperoxidase concentration. Adjusted R^2^ = 0.98.

The enzyme calibration curve in units mL^-1^ versus absorbance at 420 nm (Fig 3) establishes the relationship between the known concentrations of the analyte and the absorbance [17]. The calibration curve obtained in this work had a determination coefficient of 0.98, showing linearity similar to the curve found by [18]. This value expresses the validation of the analytical method used in this experiment, ensuring reliability in relation to the methodology used and the results found. [19] found R^2^ = 0.98 for the enzyme calibration curve when determining the lactoperoxidase concentration using TMB (3.3’,5.5’-tetramethylbenzidine) as a chromogenic agent for developing a test strip for enzymatic identification and using a color analyzer when compared to the TMB methodology by the spectrophotometric method, thus validating the method used.

### Image processing

The digital imaging devices used in this work had the purpose of capturing sections of the sample and converting them into measurable quantities of their RGB data, thereby enabling to obtain mathematical relationships between the studied variable, in this case, the enzymatic activity, and the variables which describe the system, i.e. the RGB channels [20].

This evaluation enables the study to detect the main similarity, association, and correlation patterns so that the images can subsequently be used to provide quantitative measures [12,20–23].

The primary colors in the RGB data system (red—700.0 nm; green—546.1 nm; and blue—435.8 nm) can be articulated to simulate all existing colors in the visible region spectrum [24]. In this case, univariate and multivariate calibration models can be constructed using color intensity in the RGB channels or values based on absorbance, usually using chemometric tools [20,21].

The color generated by the sample can be correlated with the concentration of the substance to be analyzed, thus presenting a semi-quantitative response in which the color provided can be compared with a previously established color gradient and the concentration of the substance in the sample can be estimated by visual comparison [22,23].

Imaging measurements are performed with the use of an image capture device, quantifying and analyzing the color intensity in the colored region. The results are then correlated with the analyte concentration using previously constructed calibration curves [12,25,26]. Then, the quantification of compounds is obtained by processing digital images using the RGB color system (Red-Green-Blue), in which its analytical response is in the visible region (~400–750 nm).

The PCA model composed of scores and loadings was used as exploratory analysis to investigate a response pattern for the lactoperoxidase enzymatic determination tested in milk samples, which are used to identify the similarity/dissimilarity between samples and evaluate the contribution size of each variable for the model, respectively [27].

Principal component analysis is one of the most widely used chemometric techniques, as it enables reducing the dimensionality of the data without losing relevant information, highlighting significant analytical signals, in addition to serving as a basis for other chemometric methods [28].

This model has the advantages of understanding and effectively visualizing data and clarifying the interrelationship between the samples and the variables studied. One of the most useful mechanisms of the PCA is that it produces a linear transformation of the data calculated by a simple multiplication of matrices using a smaller data set and the transformation is quickly calculated for the rest of the data [29].

**Fig 4:**
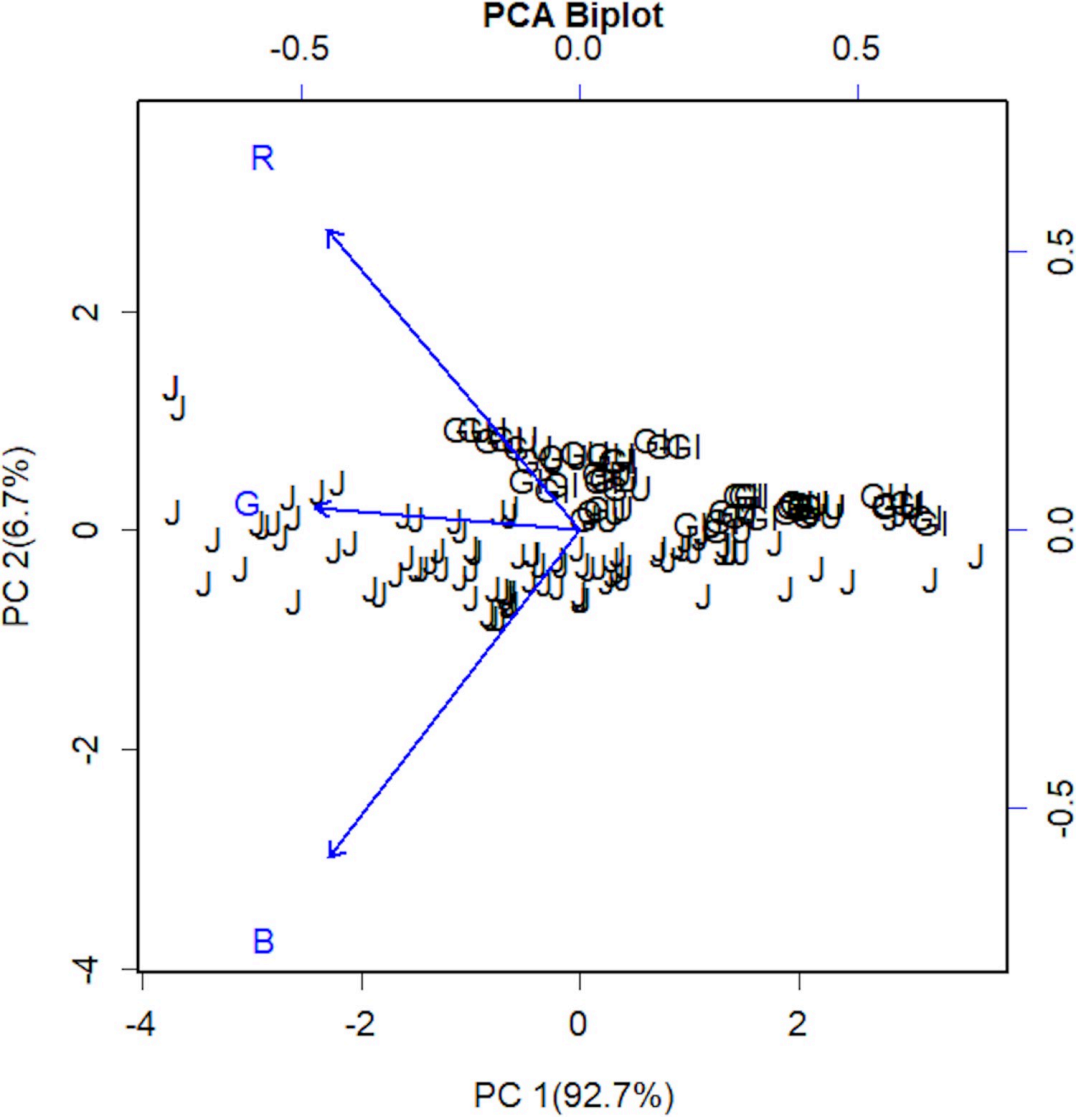
Multivariate data analysis comparing milk treated samples and RGB channels. Principal Component Analysis (PCA) model biplot of variables in Jersey (J), Gir (GI), and Guzerat (GU) milk samples with autoscaling.

The samples and RGB channels are plotted by Principal Component Analysis (Fig 4) and the biplot shows that the RGB channels increase in intensity according to the enzymatic activity (right to left). Although RGB channels had increased for all races, the Blue channel is proeminent in intensity in the Jersey samples and the Red channel in the Gir and Guzerat samples. It is concluded that all channels increase in intensity as the lactoperoxidase concentration increases, from right to left.

### Enzymatic activity calibration

**Fig 5:**
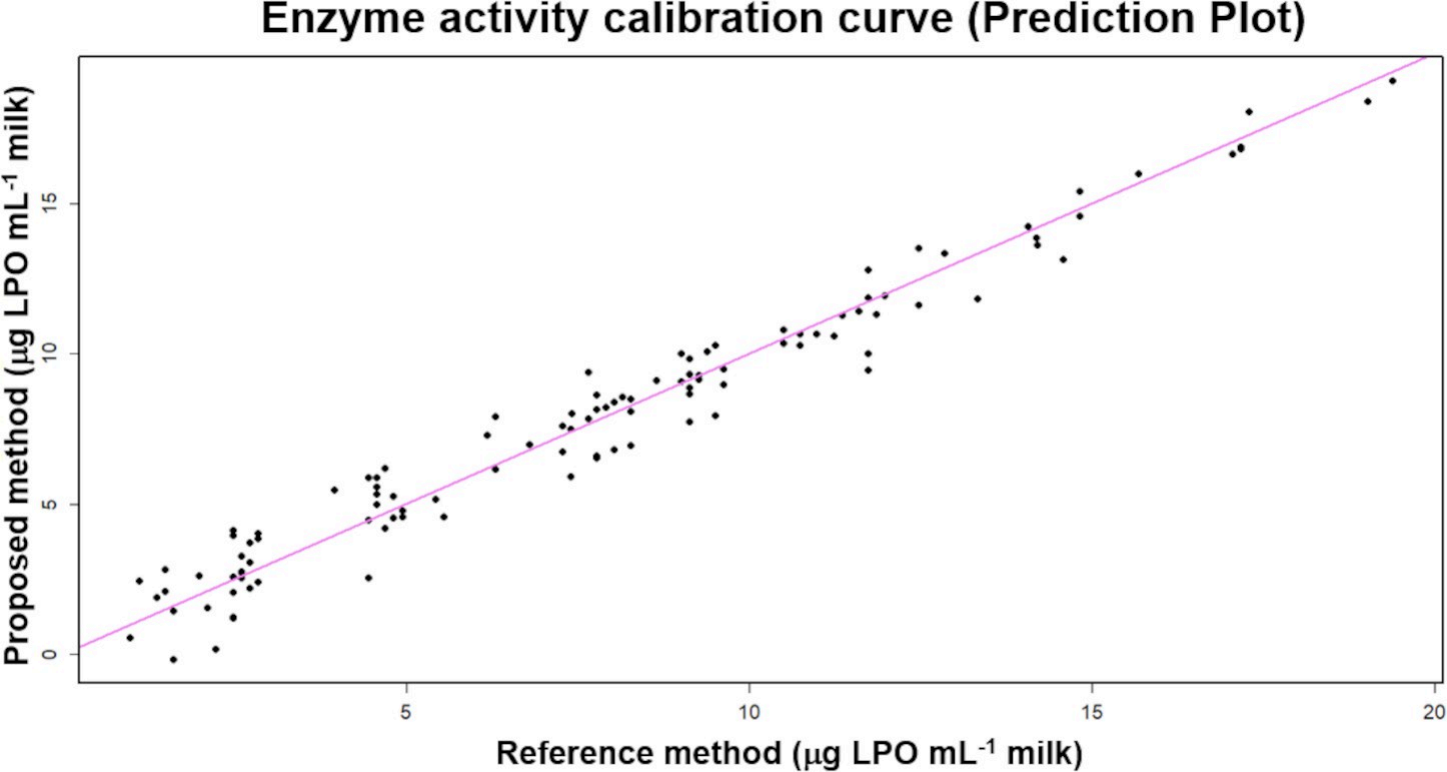
Enzyme activity calibration curve. Lactoperoxidase analysis showing the predicted activity (y-axis) vs measured activity (x-axis) and the correlation between the proposed method for quantifying lactoperoxidase and the reference method. Adjusted R^2^ = 0.96 by multiple linear regression.

The samples quantified for lactoperoxidase were subjected to multivariate regression analysis for the three RGB color channels and compared to the standard model. The results presented reasonable deviations in its responses considering that it is simple and low-cost. The coefficient of determination was 0.96 by MLR, as shown in Fig 5. Second-Order Regression was applied to the milk dataset to obtain a better adjustment, resulting in an adjusted R-squared of 0.98. It is important to emphasize that variations in the enzyme concentration may depend on the stage of the cow’s estrous cycle, feeding regime, and breed [30]. In addition, studies carried out with milk from cows and goats that presented a clinical mastitis picture showed that the lactoperoxidase enzyme undergoes modifications, increasing its activity [31]. This result suggests that enzyme activity can effectively be measured by this model and may help to identify cows with mastitis.

[32] also intended to develop a rapid colorimetric method to measure the lactoperoxidase activity based on a multiple linear regression model. The study found the R^2^ = 0.97 with the Rottenfusser method (which uses guaiacol and *p*-phenylenediamine) in comparison to the method described by [33]. The coefficient of determination was 0.98 when used the method described by [34], based on the use of ABTS.

[35] evaluated the enzymatic inactivation in goat’s milk at temperatures of 69°C, 71°C, and 73°C, and obtained R^2^ values of 0.96, 0.94, and 0.97, respectively, concluding that enzymatic stability decreases with increasing temperature.

[36] evaluated the inactivation of pathogenic bacteria in milk using the combination of an ultrasound with the lactoperoxidase system, and the observed results showed that there was a greater microbial reduction in less amplitude and time when compared to the use of ultrasound alone. The regression models obtained in this study showed a coefficient of determination ranging from 0.9991 to 0.9999 for different bacterial cultures.

### Somatic cell count calibration

**Fig 6:**
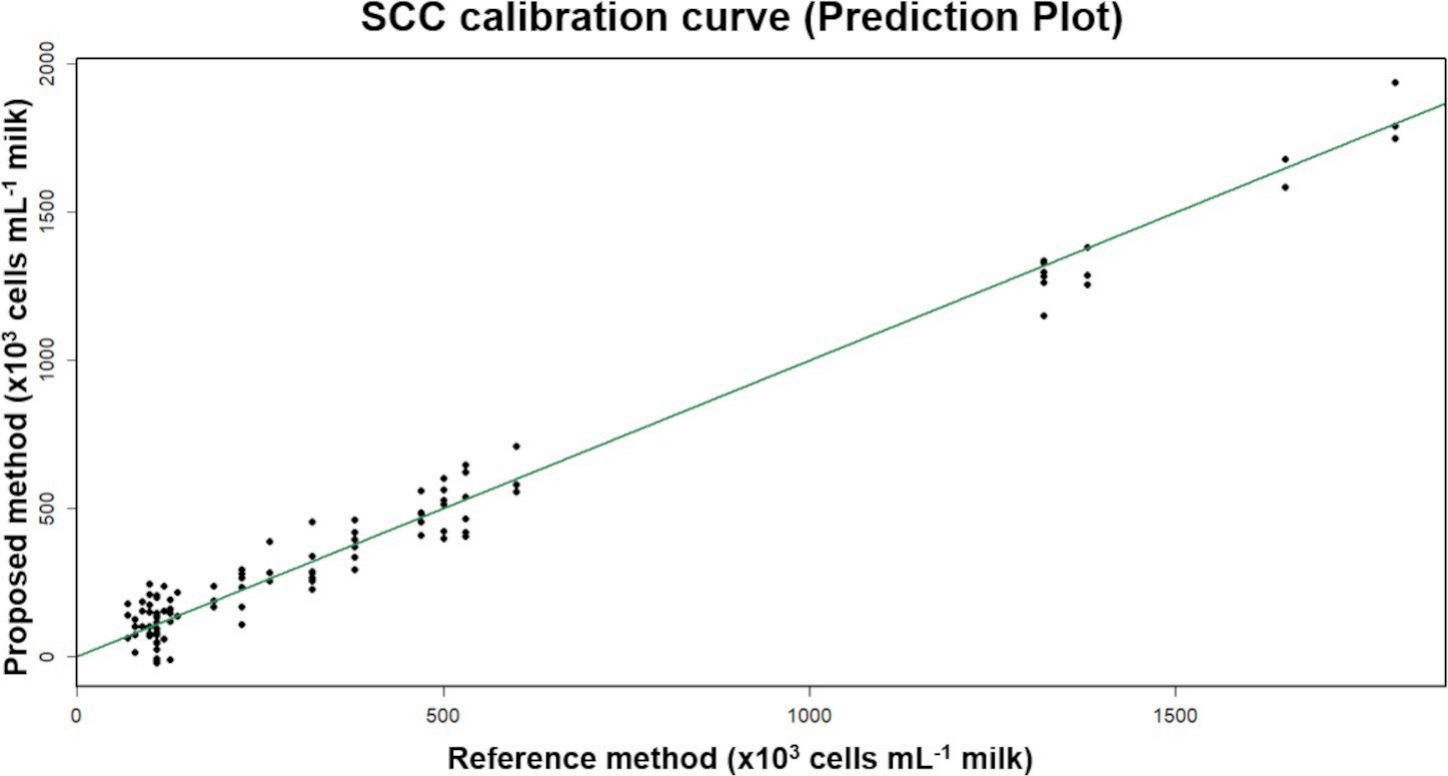
Multiple linear regression for CCS. Image processing analysis of predicted (RGB) vs. measured CCS. Adjusted R^2^ = 0.97.

The coefficient of determination was 0.97 in correlating the proposed model with the somatic cell count (SCC) experimental data by MLR, which is represented in Fig 6. The obtained result suggests that the somatic cell count can be determined by this model, indicating if the cow has subclinical mastitis, but other factors can also interfere with the SCC such as time of year, environment, animal breed, and the number of lactation days [37,38]. However, udder infection is the main factor responsible for the increase in SCC [39]. A related behavior between the predicted SCC and the measured concentrations was observed using Second-Order Regression (R^2^ = 0.99).

Considering other studies which have been conducted regarding the behavior of SCC [40], evaluated bioactive amines in mozzarella cheese (which can be harmful to the consumer) produced with milk containing different somatic cell count levels and observed that there was greater proteolysis in the cheese samples made with milk with a higher number of SCC, obtaining the R^2^ value = 0.90 for the correlation of bioactive amines and somatic cell count.

In developing an electrochemical method for determining bacterial count, Listeria monocytogenes, and somatic cell count [41], obtained the R^2^ value = 0.90 when associating the current emitted by an amperometric sensor with the amount of SCC present in raw milk.

When designing a portable somatic cell counter consisting of a camera and a miniaturized microscope [42], observed that the results for counting somatic cells in milk are compatible with the results presented by the conventional microscope, obtaining a value of R^2^ = 0.99 when comparing the association between the two methods.

## Conclusion

The use of digital images can potentially replace equipment such as the spectrophotometer for a variety of colorimetric reactions, as they are cheaper and have easy accessibility. The ease in capturing images and obtaining them with a cell phone, digital cameras, and scanners are advantages that support its use in analytical quantification.

The lactoperoxidase enzyme and the somatic cell count in milk samples from cows can be measured via images, and the enzymatic method may be used as a health indicator of the mammary gland.

Therefore, lactoperoxidase can be quantified by this method and contribute as an evaluative parameter in determining mastitis in dairy cows.

## Supporting information

S1 Fig Data that originated Fig 3. (PDF)

S2 Fig Data that originated Figs 3 and 5. (PDF)

S3 Fig Data that originated Fig 4. (PDF)
