# Supplementary figures and images for: Lactoperoxidase potential in diagnosing subclinical mastitis in cows via image processing
Source: PLoS One. 2022 Feb 17;17(2):e0263714. doi: 10.1371/journal.pone.0263714 (PMC8853571; doi:10.1371/journal.pone.0263714)

| Absorbance | Samples (microgram/mL) |
|------------|------------------------|
| 0.007      | 100                    |
| 0.024      | 200                    |
| 0.039      | 300                    |
| 0.052      | 400                    |
| 0.078      | 500                    |
| 0.098      | 600                    |
| 0.117      | 700                    |
| 0.145      | 800                    |
| 0.153      | 900                    |

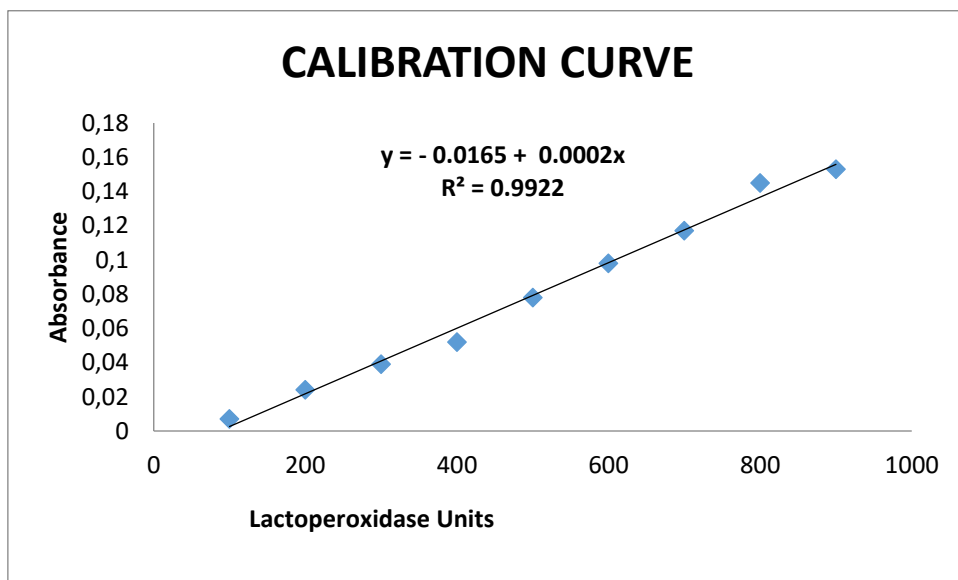

Supplement: S1 Fig — (PDF) [file pone.0263714.s001.pdf]
