# Supplementary material for: Lactoperoxidase potential in diagnosing subclinical mastitis in cows via image processing
Source: PLoS One. 2022 Feb 17;17(2):e0263714. doi: 10.1371/journal.pone.0263714 (PMC8853571; doi:10.1371/journal.pone.0263714)
Supplement: S2 Fig — (PDF) [file pone.0263714.s002.pdf]

SAMPLES (MICROGRAM/mL)	ABSORBANCE	LACTOPEROXIDASE/ML
A 100A	0.01	0.005
A 100B	0.006	0.003
A 100C	0.005	0.003
A 200A	0.027	0.013
A 200B	0.023	0.011
A 200C	0.022	0.011
A 300A	0.039	0.019
A 300B	0.038	0.018
A 300C	0.039	0.019
A 400A	0.051	0.025
A 400B	0.054	0.026
A 400C	0.051	0.025
A 500A	0.078	0.039
A500B	0.08	0.039
A 500C	0.075	0.037
A 600A	0.1	0.05
A600B	0.097	0.05
A600C	0.098	0.05
A700A	0.119	0.058
A700B	0.118	0.058
A700C	0.114	0.056
A 800A	0.143	0.07
A 800B	0.141	0.07
A 800C	0.151	0.074
A 900A	0.156	0.073
A 900B	0.149	0.071
A 900C	0.153	0.071

SAMPLES	AVERAGE ABSORBANCE	AVERAGE ENZYMATIC ACTIVITY
100	0.007	0.004
200	0.024	0.012
300	0.039	0.019
400	0.052	0.025
500	0.078	0.038
600	0.098	0.05
700	0.117	0.057
800	0.145	0.071
900	0.153	0.072
