# Supplementary material for: Lactoperoxidase potential in diagnosing subclinical mastitis in cows via image processing
Source: PLoS One. 2022 Feb 17;17(2):e0263714. doi: 10.1371/journal.pone.0263714 (PMC8853571; doi:10.1371/journal.pone.0263714)
Supplement: S3 Fig — (PDF) [file pone.0263714.s003.pdf]

Breed	R	G	B	
GI05A	0.260	0.530	0.652	
GI05B	0.255	0.518	0.651	
GI05C	0.243	0.520	0.629	
GI06A	0.257	0.567	0.710	
GI06B	0.256	0.564	0.678	
GI06C	0.260	0.560	0.684	
GI07A	0.181	0.445	0.603	
GI07B	0.179	0.444	0.606	
GI07C	0.184	0.449	0.613	
GI08A	0.190	0.472	0.641	
GI08B	0.203	0.488	0.671	
GI08C	0.198	0.484	0.652	
GI14A	0.136	0.375	0.538	
GI14B	0.148	0.384	0.539	
GI14C	0.131	0.371	0.539	
GI15A	0.206	0.482	0.637	
GI15B	0.204	0.472	0.632	
GI15C	0.204	0.478	0.634	
GI16A	0.264	0.602	0.732	
GI16B	0.267	0.602	0.744	
GI16C	0.279	0.619	0.751	
GU01A		0.206	0.513	0.684
GU01B		0.193	0.500	0.668
GU01C		0.200	0.493	0.651
GU02A		0.294	0.615	0.727
GU02B		0.286	0.605	0.733
GU02C		0.309	0.625	0.743
GU03A		0.178	0.457	0.598
GU03B		0.177	0.460	0.611

GU03C		0.177	0.460	0.595
GU04A		0.164	0.384	0.555
GU04B		0.154	0.380	0.552
GU04C		0.152	0.387	0.553
GU09A		0.316	0.643	0.740
GU09B		0.303	0.623	0.729
GU09C		0.321	0.653	0.749
GU10A		0.249	0.546	0.704
GU10B		0.261	0.556	0.706
GU10C		0.255	0.556	0.703
GU11A		0.266	0.559	0.686
GU11B		0.276	0.574	0.701
GU11C		0.284	0.579	0.722
GU12A		0.245	0.558	0.730
GU12B		0.245	0.570	0.745
GU12C		0.246	0.562	0.738
GU13A		0.169	0.455	0.587
GU13B		0.167	0.454	0.596
GU13C		0.168	0.455	0.582
J01A	0.343	0.770	0.101	
J01B	0.325	0.703	0.102	
J01C	0.357	0.770	0.105	
J02A	0.226	0.549	0.773	
J02B	0.223	0.541	0.789	
J02C	0.256	0.632	0.892	
J03A	0.180	0.497	0.745	
J03B	0.154	0.451	0.687	
J03C	0.156	0.422	0.657	
J04A	0.219	0.507	0.743	
J04B	0.216	0.538	0.785	

J04C	0.222	0.538	0.783
J05A	0.205	0.518	0.705
J05B	0.203	0.527	0.714
J05C	0.222	0.557	0.759
J07A	0.241	0.609	0.894
J07B	0.242	0.619	0.894
J07C	0.245	0.618	0.896
J08A	0.264	0.648	0.877
J08B	0.266	0.638	0.864
J08C	0.258	0.602	0.830
J09A	0.226	0.557	0.827
J09B	0.220	0.561	0.825
J09C	0.218	0.570	0.825
J10A	0.139	0.406	0.647
J10B	0.111	0.367	0.590
J10C	0.104	0.342	0.541
J11A	0.256	0.594	0.876
J11B	0.250	0.598	0.869
J11C	0.253	0.595	0.874
J12A	0.183	0.511	0.684
J12B	0.186	0.507	0.679
J12C	0.198	0.513	0.708
J13A	0.354	0.769	0.958
J13B	0.368	0.777	0.101
J13C	0.334	0.770	0.946
J14A	0.302	0.675	0.856
J14B	0.383	0.827	0.997
J14C	0.308	0.683	0.862
J16A	0.232	0.570	0.794
J16B	0.245	0.576	0.745

J16C	0.238	0.578	0.781
J17A	0.144	0.402	0.558
J17B	0.173	0.466	0.652
J17C	0.144	0.394	0.551
J18A	0.279	0.675	0.891
J18B	0.286	0.691	0.941
J18C	0.282	0.675	0.895
J19A	0.227	0.554	0.787
J19B	0.220	0.538	0.773
J19C	0.223	0.540	0.801
J20A	0.246	0.605	0.873
J20B	0.243	0.602	0.875
J20C	0.244	0.602	0.879
J21A	0.454	0.779	0.926
J21B	0.443	0.775	0.936
J21C	0.452	0.777	0.926
J22A	0.202	0.508	0.695
J22B	0.206	0.500	0.693
J22C	0.205	0.511	0.717
J23A	0.348	0.773	0.953
J23B	0.342	0.778	0.937
J23C	0.339	0.762	0.924
J24A	0.352	0.719	0.903
J24B	0.360	0.735	0.926
J24C	0.348	0.713	0.881
J25A	0.252	0.603	0.816
J25B	0.241	0.587	0.804
J25C	0.244	0.598	0.839
J26A	0.230	0.560	0.795
J26B	0.200	0.534	0.728

J26C	0.209	0.533	0.735
J27A	0.247	0.592	0.821
J27B	0.237	0.586	0.804
J27C	0.253	0.598	0.816
J28A	0.288	0.705	0.938
J28B	0.286	0.692	0.885
J28C	0.282	0.699	0.906
J29A	0.280	0.641	0.890
J29B	0.275	0.634	0.850
J29C	0.273	0.633	0.851
J30A	0.319	0.703	0.921
J30B	0.326	0.699	0.945
J30C	0.293	0.644	0.839
J31A	0.186	0.473	0.687
J31B	0.189	0.478	0.694
J31C	0.190	0.482	0.694
J32A	0.286	0.646	0.840
J32B	0.290	0.641	0.878
J32C	0.287	0.647	0.891
J33A	0.290	0.597	0.815
J33B	0.282	0.603	0.798
J33C	0.282	0.596	0.809
J34A	0.234	0.598	0.829
J34B	0.227	0.596	0.821
J34C	0.250	0.626	0.866
